# Reply to Sussman et al. Comment on “Svarch-Pérez et al. Methods for a Non-Targeted Qualitative Analysis and Quantification of Benzene, Toluene, and Xylenes by Gas Chromatography-Mass Spectrometry of E-Liquids and Aerosols in Commercially Available Electronic Cigarettes in Mexico. *Int. J. Environ. Res. Public Health* 2024, *21*, 1308”

**DOI:** 10.3390/ijerph22071050

**Published:** 2025-06-30

**Authors:** Alejandro Svarch-Pérez, María Vanessa Paz-González, Carlota Ruiz-Juárez, Juan C. Olvera-Chacón, Angelina Larios-Solís, Santiago Castro-Gaytán, Eugenia Aldeco-Pérez, Jorge Carlos Alcocer-Varela

**Affiliations:** 1 Federal Commission for the Protection Against Sanitary Risks, Oklahoma 14, Col. Nápoles, D.T. Benito Juárez, Mexico City 03810, Mexico; 2 Center for Research and Technological Development in Electrochemistry, S.C., Parque Tecnológico Querétaro SN, Pedro Escobedo, Querétaro 76703, Mexico; 3 Health Secretary, Homero 213, Piso 15 Col. Chapultepec Morales, D.T. Miguel Hidalgo, Mexico City 11570, Mexico

The paper titled “Methods for a Non-Targeted Qualitative Analysis and Quantification of Benzene, Toluene, and Xylenes by Gas Chromatography-Mass Spectrometry of E-Liquids and Aerosols in Commercially Available Electronic Cigarettes in Mexico” was submitted in compliance with all the requirements of the editorial and journal in question, as evidenced by the dates and the respective backup documentation. This work was conducted following the analytical procedures outlined for the detection and quantification of chemical compounds. The files that attest to this were posted on the journal platform along with the Supplementary Information files. 

The findings of our work are as follows: e-liquids and aerosols obtained from commercially available e-cigarettes were analyzed by a self-developed procedure using GC-MS chromatography. The results showed that the e-liquids have between 13 and 71 compounds, and the aerosol phases of the same e-liquids have between 73 and 167 compounds. BTXs (benzene, toluene, and xylenes) were quantified in e-liquid and aerosol samples.

The most valuable result was that BTXs were found in several samples, in addition to a list of other dangerous chemicals. It is known that these kinds of compounds have elevated toxicity, induce cancer, and cause central nervous system and lung damage. Our analysis was conducted with an easy-to-use experimental methodology, so that any gas chromatography facility could perform it, without the use of special smoking machines. Below, we will answer the points included in the first part of the comment.

1. To avoid controversies, and to provide the easiest understanding of concentrations, we decided to present the data in ppm, including the mean concentration found in cigarettes, permissible exposure limit (PEL), short-term exposure limit (STEL), and experimental concentration values, keeping in mind that the PEL is an 8 h limit concentration of a substance that a worker can be exposed to under Occupational Safety and Health Administration (OSHA) regulations, and the STEL is a 15 min average exposure concentration during a workday. PELs are regulatory limits on exposure designed to prevent harm and do not represent chemical toxicity [1]. A new version of Table 3, Table 4 and Table 5 is presented at the end of this document.

2, 3. In their comment [3], the authors mention an erroneous comparison of BTX results with the OSHA PEL and STEL. They also propose an estimation assuming the average values found in their own surveys (volume, puffs, etc.), which cannot be representative of our study. Our study was just a general and rough comparison with the PEL and STEL, although we are aware that specific reference values of maximum permitted limits for ENDSs (electronic nicotine delivery systems) in e-liquid or aerosol phases do not exist, and neither do local information about ENDS user habits and real consumption. The reviewers proposed PEL and STEL and agreed on the second revision of the manuscript, since these limits are the most suitable parameters for comparison purposes and are also found in some previous reports [2]. Unfortunately, none of these exposure limits envisage the risk and real damage to ENDS consumers, whether it is children, young people, or adults that are the direct or indirect end users. The limitations of PEL/STEL values are well identified: exposure limits with long-term (chronic) effects are not contemplated; the limits are not the same for everybody (depending on physical activity and susceptibility); chemical interactions are not considered; and the most important fact is that the data refers to work in environments where particles are diluted in the air and can pass through the worker’s respiratory system [4]. The action of vaping is not comparable to just taking a breath of polluted air, since it is an action that directly introduces a mixture of aerosolized substances into the respiratory system through the mouth and that could be performed, depending on the user’s habits and preferences, as a mouth-to-lung or direct-to-lung action [5,6,7,8]. No examples of data interpretation or data treatment as indicated in the comment were found in the literature. The dose consumed and the approximation of real exposure, although they are topics of interest, are beyond the scope of our first manuscript.

The most viable strategy for accurate risk assessment calculation could be the use of the Consumer Exposure Model (CEM) developed by the U.S. EPA [9]. This model is employed to calculate the risk assessment using the Acute Dose Rate (ADR), Chronic Dose Rate (CDR), and Lifetime Average Daily Dose (LADD) for firsthand and secondhand exposure scenarios. The cancer risk for each compound is also considered; however, it was not within the scope of our publication.

4. Regarding the general assumptions in the comment, we find that conventions like average inhalation rate, or 5 mL of vaping daily volume, could be an uncertain approximation in a population where most of the consumers are adolescents (2.6% of the population in Mexico) [10]. Moreover, the inhalation of ENDS products as well as smoking, implies more inhalation volume, as it is deeper and more direct than simple breathing (depending on the user’s health status, altitude, gender, body size, age, lung capacity, experience, etc., in addition to the features and quality of the specific ENDS device’s design and the materials used in its construction, such as plastics, electronic components, resistors, and batteries).

Furthermore, vaping, like smoking regularly, is a social habit, during which the person who is vaping/smoking inhales extra particles from the environment they are vaping/smoking in. The habits of people who vape are not limited to an eight-hour workday or a forty-hour week [11].

We then made a general comparison, which was accepted during the second review process of the manuscript. Besides PEL and STEL, we also chose a mean concentration of BTXs found in combustible cigarettes, where only the final concentrations are compared.

We know this is a complex system with many variables. Please consider our values to be an estimate; we cannot compare the direct level of toxicity caused by these types of devices in humans with exact data. Years of studies and thousands of data points are needed to establish the most appropriate reference parameters for the complex system under discussion.

We used PEL and STEL as references, since a reviewer suggested it. A direct comparison of these values was performed in the past, considering the concentration expelled by combustible cigarettes [2]. We are aware that this kind of comparison is not totally accurate, so it must be considered a general and rough estimation and should not be taken as a toxicological reference.

The approximations proposed in the comment do not consider the user’s lung volume, the altitude at which the product is consumed, the particle size, and other factors that affect this phenomenon. One extra point to consider is that under normal conditions, BTX compounds are present in the ambient atmospheric air, for example, in Mexico City, a highly densely populated city [12]. If we were to be strict, we would also need to account for the additive toxic concentration, as well as the initial BTXs in the corresponding urban atmosphere (where most ENDS consumers are located). It is interesting to think about the different sources of BTXs found in a city environment: vehicles moving slowly on main avenues, public buses, drivers smoking, industrial activity sources, kitchens, etc.

Finally, it is important to highlight the objective of our work: to quantify BTX compounds and to qualitatively assess the contents of e-liquids and their corresponding aerosols present in ENDS devices.

Below are our answers to the analytical methodology paragraph:

1. The tables (3, 4, and 5 in manuscript) had a typographical error, so we include a corrected version here. The data are presented in ppm units, considering the suggestions.
2. Regarding the recommended CORESTA method, ISO 7210:2013 and related methods define the generation and collection of aerosols coming from a smoking machine equipped with an e-cigarette holder, aerosol traps, and filter disk, among other components. Unfortunately, we do not have a smoking machine, which is why we decided to sample the aerosols using Tenax tubes, a nitrogen flow, and a vacuum pump to extract them. It is also stated in the CORESTA method design that “this standard can be used as a mechanism for comparison of products based on aerosol deliveries, it by no means represents the only strategy for the collection of e-vapor aerosol” [13,14,15].

As an example of a similar sampling method, we found previous reports [16,17] which used three-puff sampling “to avoid the buildup of contamination in the ENDS device, which might lead to higher values of VOCs” in a similar study on benzene quantification in ENDS liquid mixtures. With this in mind, we consider our sampling method a good choice, since when it comes to more concentrated sampling, dilutions of up to 20 times, for example, have to be executed in order to facilitate GC analysis, so a two-puff sample was just our first approach, and it worked fine. In addition to searching for BTXs in the sample, it has been reported that vaping can generate toxic compounds such as benzene, so using two puffs seemed appropriate to prevent the coil from overheating and to observe what other compounds could be generated [18].

3, 4, 5. Regarding our analytical procedures, there were just two: purge and trap and thermal desorption. The purge and trap procedure consists of two types of solvent extraction (see Figure 2 in the manuscript). Regarding the methodologies, just to clarify, two different pieces of equipment were used, each one having its own column of the same type and each one quantified separately (the details are in the manuscript and Supporting Information). Retention times are all included in the extra file uploaded as Supporting Information, assessed by reviewers. Internal standards are described in the Supporting Information, with catalog numbers and brands. It is also important to mention that our working team has experience in BTX chromatography quantification for both liquid and gas phases. Regarding chromatographic analysis, we submitted the calibration curves, chromatograms, and the required supporting documentation alongside the manuscript, demonstrating the validity of the procedures and data processing.

In conclusion, this work should be seen as a starting point for local vaping research. From here, we can suggest changes, improvements, and new procedures that will lead to the creation of national regulations that can be used as a reference. There are plans to continue publishing on this topic, including on toxicity and long-term user monitoring, to assess the damage caused. It is certain, though, that finding even a small concentration of BTXs in deep-inhalation devices such as ENDSs is not desirable, since benzene is part of group 1 and toluene of group 3, according to the International Agency for Research on Cancer; also, BTXs are considered hazardous pollutants, as listed by the U.S.EPA. 

Benzene, toluene, xylene, acrolein and its derivatives, and nicotine (detected in some of the samples, and according to the label, nicotine until 50 mg/mL) all have adverse health effects, including acute irritation and inflammation of the respiratory system, with varying consequences depending on the individual. They may exhibit additive or synergistic effects, which underscores the relevance of comparing exposure levels with PEL and STEL to support informed decision-making regarding the use of ENDS.

Based on these findings, the presence of BTXs, even at very low concentrations, should be taken very seriously, since harm to an individual may be due to the length of time the product is inhaled and to the combination of substances that make up the mixture, among other factors.

We need more realistic data tailored to the population most susceptible to harm, including inhalation patterns, frequency of use, lung volume, health status, device type, quality of the e-liquid contained, chronic exposure, and other factors. National surveys with a local scope are needed, so that we can have valid data to compare commercially available products in a determined population, considering that children and adolescents are a vulnerable segment of the population, following WHO recommendations [19].

As an example, on 1 June 2025, England banned the sale of single-use e-cigarettes with and without nicotine [20], as their design, colors, flavors, advertising, and price encourage children and adolescents to use them. This explains the importance of continuing to conduct studies on e-cigarettes in unregulated markets, because this is where young people can access them, and the quality of both the e-liquid and the device can be diminished, endangering this segment of the population.

Table 3, Table 4 and Table 5 include values in ppm units, modified from reference [21].

## Figures and Tables

**Table 3:** Benzene concentrations in e-liquids and aerosols and reference parameters.

Benzene					
Reference Concentration Parameters			Experimental Concentration Values		
Mean concentration found in cigarettes [2] ppm	Permissible exposure limit (PEL) * ppm	Short-term exposure limit (STEL) # ppm	E-liquid in water extraction ppm	E-liquid in methanol extraction ppm	Aerosol estimated ppm
-		-	0.072 min	0.180 min	0.517 min
1.295	1	5	0.680 max	0.817 max	9.562 max

* PEL: Maximum permitted 8 h time-weighted average concentration of an airborne contaminant [1]. # STEL: A 15 min time-weighted average exposure which is not to be exceeded at any time during a workday [1]. Min = minimum obtained value. Max = maximum obtained value.

**Table 4:** Toluene concentrations in e-liquids and aerosols and reference parameters.

	Toluene				
	Reference Concentration Parameters		Experimental Concentration Values		
Mean concentration found in cigarettes [2] ppm	Permissible exposure limit (PEL) * ppm	Short-term exposure limit (STEL) # ppm	E-liquid in water extraction ppm	E-liquid in methanol extraction ppm	Aerosol estimated ppm
-	-	-	0.088 min	0.278 min	0.115 min
7.691	10	150	2.562 max	4.355 max	130.618 max

* PEL: Maximum permitted 8 h time-weighted average concentration of an airborne contaminant [1]. # STEL: A 15 min time-weighted average exposure which is not to be exceeded at any time during a workday [1]. Min = minimum obtained value. Max = maximum obtained value.

**Table 5:** Xylene concentrations in e-liquids and aerosols and reference parameters.

	Xylenes				
	Reference Concentration Parameters		Experimental Concentration Values		
Mean concentration found in cigarettes [2] ppm	Permissible exposure limit (PEL) * ppm	Short-term exposure limit (STEL) # ppm	E-liquid in water extraction ppm	E-liquid in methanol extraction ppm	Aerosol estimated ppm
-	-	-	0.163 min	0.778 min	0.799 min
1.079 ≠	100	150	17.981 max	15.429 max	165.555 max

* PEL: Maximum permitted 8 h time-weighted average concentration of an airborne contaminant [1]. # STEL: A 15 min time-weighted average exposure which is not to be exceeded at any time during a workday [1]. ≠ m and p-xylenes were quantified. Min = minimum obtained value. Max = maximum obtained value.
